# High-dose olanzapine in treatment resistant schizophrenia. A case report and literature review

**DOI:** 10.1192/bjo.2021.335

**Published:** 2021-06-18

**Authors:** Ciara Clarke, Clodagh Rushe, Fintan Byrne

**Affiliations:** Mayo University Hospital

## Abstract

**Objective:**

We report a case of a 58-year-old gentleman who was hospitalised intermittently for one year due to treatment resistant schizophrenia. Prior to hospitalisation he had been prescribed standard antipsychotics for decades without full resolution of positive psychotic symptoms. During his final admission lasting six months he was guarded, suspicious, irritable, constantly paced the corridor and displayed thought block and paranoid persecutory delusions. He would not enter the assessment room or allow any blood or ECG monitoring, however, he was compliant with oral medication. He was successfully treated with high dose olanzapine (40mg/day) and was discharged to the community. The aim of this study is to bring awareness and add to the body of evidence for the use of high-dose olanzapine in patients with treatment resistant schizophrenia in whom a trial of clozapine is not possible.

**Case report:**

The patient gave written consent for this case report to be written and presented. An extensive literature review was performed and key papers were identified. Discussion focuses on the key areas in the literature.

**Discussion:**

This case demonstrates that high-dose olanzapine can be used effectively as an alternative to clozapine in treatment resistant schizophrenia.

**Conclusion:**

This case highlights the need for further evaluation of high-dose olanzapine as an alternative to clozapine in patients with treatment-resistant schizophrenia.

